# EasyCodeML: A visual tool for analysis of selection using CodeML

**DOI:** 10.1002/ece3.5015

**Published:** 2019-03-01

**Authors:** Fangluan Gao, Chengjie Chen, Daej A. Arab, Zhenguo Du, Yehua He, Simon Y. W. Ho

**Affiliations:** ^1^ Fujian Key Laboratory of Plant Virology, Institute of Plant Virology Fujian Agriculture and Forestry University Fuzhou China; ^2^ School of Life and Environmental Sciences University of Sydney Sydney New South Wales Australia; ^3^ College of Horticulture South China Agricultural University Guangzhou China

**Keywords:** CodeML, codon‐based models, likelihood‐ratio test, molecular evolution, positive selection

## Abstract

The genomic signatures of positive selection and evolutionary constraints can be detected by analyses of nucleotide sequences. One of the most widely used programs for this purpose is CodeML, part of the PAML package. Although a number of bioinformatics tools have been developed to facilitate the use of CodeML, these have various limitations. Here, we present a wrapper tool named EasyCodeML that provides a user‐friendly graphical interface for using CodeML. EasyCodeML has a custom running mode in which parameters can be adjusted to meet different requirements. It also offers a preset running mode in which an evolutionary analysis pipeline and publication‐quality tables can be exported by a single click. EasyCodeML allows visualized, interactive tree labelling, which greatly simplifies the use of the branch, branch‐site, and clade models of selection. The program allows comparison of major codon‐based models for analyses of selection. EasyCodeML is a stand‐alone package that is supported in Windows, Mac, and Linux operating systems, and is freely available at https://github.com/BioEasy/EasyCodeML.

## 1 INTRODUCTION

Advances in high‐throughput sequencing technologies have led to an unprecedented wealth of genome‐scale data for evolutionary analysis. These data offer valuable opportunities for investigating the effects of positive selection and constraints on genomic evolution. Although a range of bioinformatics tools and resources are readily available for using codon‐based models of evolution (Pond, Frost, & Muse, 2005; Stern et al., 2007; Valle et al., 2014; Zhang, Wang, Long, & Fan, 2013), the CodeML program in the PAML package (Yang, 2007) is among the most widely used.

One method of testing for selection is to compute *ω*, the ratio of nonsynonymous to synonymous substitution rates. Under the assumption of neutral evolution, *ω* is expected to have a value of 1. Positive and purifying (negative) selection are indicated when *ω* > 1 and *ω* < 1, respectively (Nei & Gojobori, 1986). Several different models have been implemented in CodeML, varying in terms of their assumptions about how *ω* varies across the sequence (site models) or across branches of the phylogeny (branch models; Yang, 2007).

Site models can be used to identify positively selected sites in a multiple sequence alignment (Yang & Nielsen, 2002). They employ different site‐class‐specific models, all of which assume that the *ω* ratio is the same across branches of the phylogeny but different among sites in the alignment. These codon substitution models are: M0 (one‐ratio), M1a (nearly neutral), M2a (positive selection), M3 (discrete), M7 (beta), M8 (beta and *ω* > 1) and M8a (beta and *ω* = 1). The fit of these models to the sequence data can be compared using likelihood‐ratio tests. Support for positive selection can be identified if M2a provides a better fit than M1a, or if M8 provides a better fit than M7 or M8a (Yang, Nielsen, Goldman, & Pedersen, 2000). The M8–M7 comparison offers a very stringent test of positive selection (Anisimova, Bielawski, & Yang, 2001), but the M8–M8a comparison has seen growing use because it yields fewer false positives (Swanson, Nielsen, & Yang, 2003; Wong, Yang, Goldman, & Nielsen, 2004).

Branch models can be used to test whether there are significant differences in *ω* among branches of the tree (Yang & Nielsen, 1998, 2002). There are three branch models in CodeML, including a free‐ratio model allowing an independent *ω* for each branch in the tree, a one‐ratio model (M0) assuming that *ω* has been constant throughout the tree, and a two‐ratio model assuming that specific branches have an *ω* that differs from that throughout the rest of the tree (Yang, 1998). Pairwise comparisons of these models can be performed using likelihood‐ratio tests (Anisimova et al., 2001).

Models with heterogeneous *ω* across sites and across branches can be combined in the form of branch‐site models. These models can be used to identify signals of episodic selection occurring along a specified branch after gene duplication (Yang & Nielsen, 2002; Zhang, Nielsen, & Yang, 2005). A branch‐site model that allows positive selection along specified branches (Model A) can be compared against a null model (Model A_null_) that allows neutral evolution and negative selection (Zhang et al., 2005).

Clade models allow differences in site‐specific selective constraints among clades in the tree (Bielawski & Yang, 2004; Forsberg & Christiansen, 2003). The model C (CmC) estimates a separate *ω* ratio for each of two or more clades and is compared against a null model 2a_rel (M2a_rel) in which *ω* is fixed among clades (Weadick & Chang, 2012).

If a likelihood‐ratio test yields a significant result for any of the pairwise comparisons of codon models, the Bayes empirical Bayes (BEB) method (Yang, Wong, & Nielsen, 2005) can then be used to identify amino acid residues that have potentially evolved under selection. The standard threshold for identifying amino acid sites under selection is a posterior probability of 0.95 (Scheffler & Seoighe, 2005).

The use of CodeML is controlled by variables listed in a control file, in which numerical optimization parameters can be modified to perform evolutionary analysis using a chosen codon model. The control file can be daunting for new users of CodeML. For this reason, several computer programs have been developed with the purpose of providing a more user‐friendly interface for CodeML (Table 1). However, these programs have various limitations, such as complex configuration procedures or a reduced set of codon models. For example, two recently released packages, IDEA (Interactive Display for Evolutionary Analyses; Egan et al., 2008) and IMPACT_S (Integrated Multiprogram Platform to Analyze and Combine Tests of Selection; Maldonado, et al., 2014), provide a graphical user interface but only implement three pairs of site models (M0 vs. M3, M1a vs. M2a and M7 vs. M8). Xu and Yang (2013) developed a graphical user interface for PAML named pamlX, but the complex parameter settings for CodeML still remained challenging for users. Notably, the foreground and background branches of the phylogeny must be specified (Yang & Nielsen, 2002). None of the available tools allows user‐friendly labelling of branches or nodes in the tree by one click (Table 1).

**Table 1:** Comparison of features in EasyCodeML and other tools

Key features	IDEA	pamlX	IMPACT_S	LMAP^b^	BlastPhyMe^c^	EasyCodeML
Supported codon models						
Branch model	×	✓	×	✓	✓	✓
Branch‐site model	×	✓	×	✓	✓	✓
Site model	✓^a^	✓	✓^a^	✓	✓	✓
Clade model	×	✓	×	✓	✓	✓
LRT automatically performed	×	×	✓	✓	✓	✓
Visual labelling of tree by one click	×	×	×	×	×	✓
Customizing control files	×	✓	✓	✓	×	✓
Exporting preformatted table	×	×	×	✓	✓	✓
Multithreading	×	×	×	✓	✓	✓
Drag‐and‐drop functionality	×	✓	×	×	×	✓

^a^Only a few codon‐based models available. ^b^Maldonado et al, 2016, https://doi.org/10.1186/s12859-016-1204-5. ^c^Schott et al, 2016, http://dx.doi.org/10.1101/059881.

Here, we describe EasyCodeML, a program that provides a user‐friendly interface for setting up complex analyses of selection in CodeML. In addition to a custom mode in which all parameters can be adjusted to meet the requirements of the user, EasyCodeML offers a preset mode that allows the construction of a pipeline from input to output (Supporting information Figure S1).

## 2 IMPLEMENTATION

**Figure 1:**
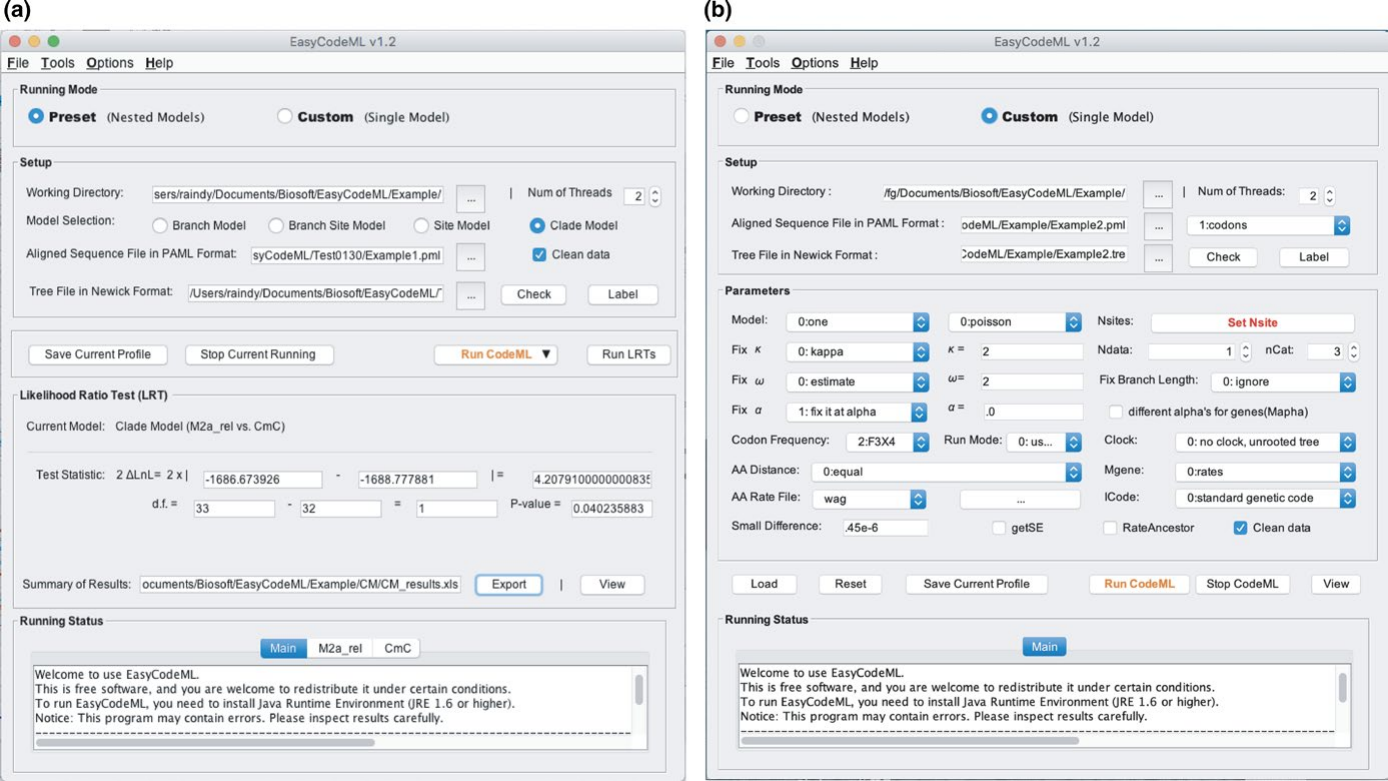
Screenshot of the main interface of EasyCodeML under the (a) preset and (b) custom running modes. In the preset mode, all key parameters of the nested models are built‐in and there is a pipeline from data input to the output of results. In the custom mode, the parameters of any codon‐based model can be modified to meet the requirements of the user

EasyCodeML provides two different running modes. The first is the preset mode (Figure 1a), in which all key parameters of the nested models are built‐in and which has pipelines for the selection analyses (Table 2). The nested models include the site models (M0 vs. M3, M1a vs. M2a, and M7 vs. M8), branch models (M0 vs. two‐ratio model), branch‐site models (Model A_null_ vs. Model A), and clade models (M2a_rel vs. CmC). The default settings in the control files for these pairs of nested models are given in Supporting information Tables S1–S4.

**Table 2:** Codon‐based models available in EasyCodeML

Codon‐based models	Running mode		Nested models (null vs. alternative)
	Preset	Custom	
Site models			
M0 (one‐ratio)	✓	✓	M3 versus M0 a
M1 (nearly neutral)	✓	✓	M1a versus M2a
M2a (positive selection)	✓	✓	M7 versus M8
M3 (discrete)	✓	✓	M8a versus M8
M7 (beta)	✓	✓	
M8 (beta and *ω* > 1)	✓	✓	
M8a (beta and *ω* = 1)	✓	✓	
Branch model			
One‐ratio model (M0)	✓	✓	M0 versus BM
Two‐ratio model (BM)	✓	✓	M0 versus FM
Free‐ratio model (FM)	×	✓	
Branch‐site models			
Model A_null_	✓	✓	Model A_null_ versus Model A
Model A	✓	✓	
Clade models			
M2a_rel	✓	✓	M2a_rel versus CmC
CmC	✓	✓	

a The M0–M3 comparison does not allow detection of positive selection.

The second running mode is the custom mode for experienced users (Figure 1b). As with pamlX, the parameters for any codon‐based model can be modified to meet different requirements. Notably, a utility named “control file viewer” is integrated in the custom running mode in EasyCodeML. This includes all of the described codon‐based models, with preoptimized parameters.

**Figure 2:**
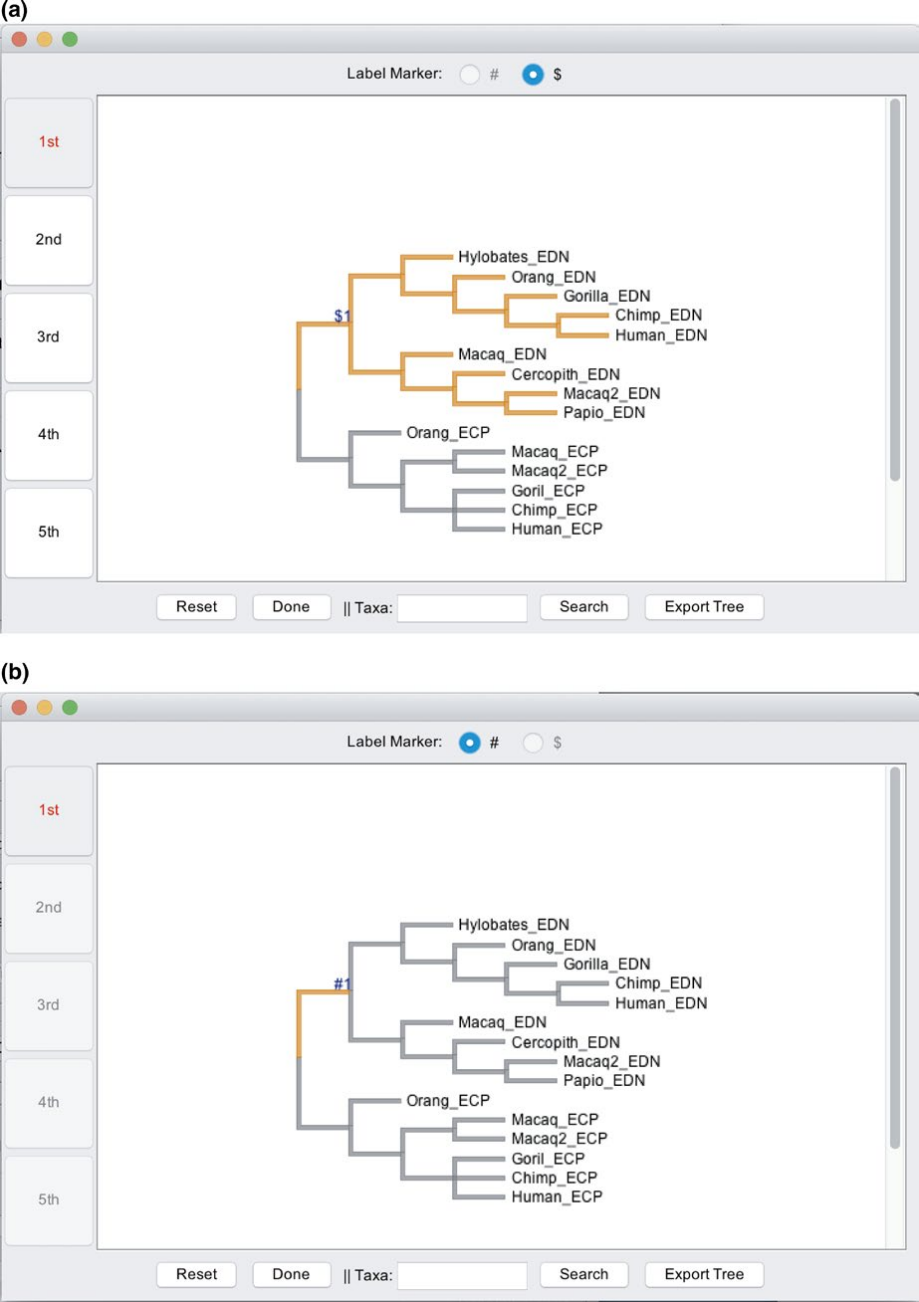
Labelling branches in a tree for the branch‐related models can be done in a simple and intuitive way for the (a) clade models and (b) branch and branch‐site models

When using the models involving heterogeneous *ω* among branches, it can be a challenging task to label branches or nodes in the phylogenetic tree. Performing this task using a text editor is difficult and prone to error. EasyCodeML provides a graphical interface that allows the labelling of branches and nodes to be done in a visualized, interactive way (Figure 2).

**Figure 3:**
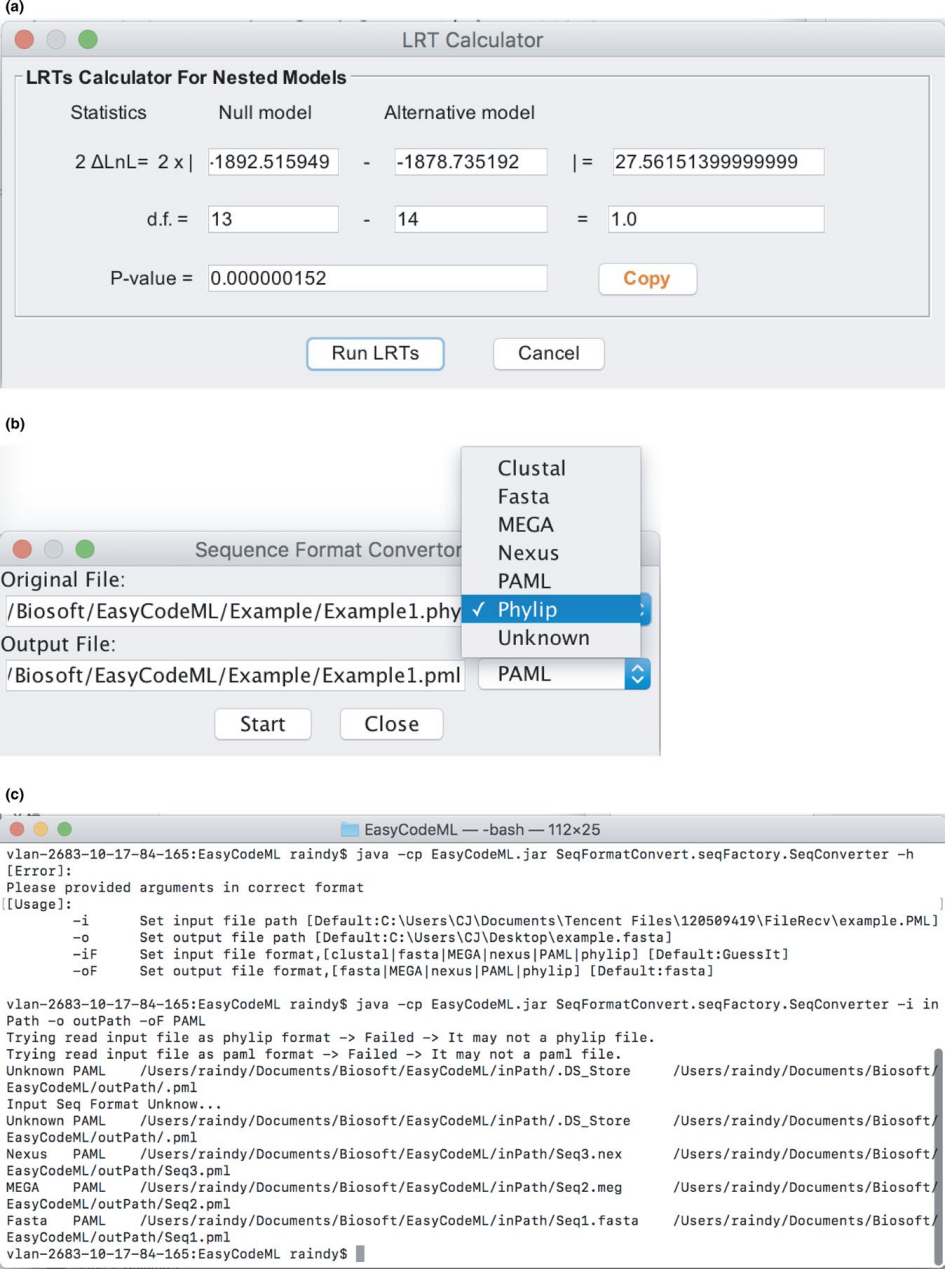
Two utilities available in EasyCodeML: (a) the LRT calculator, and Seqformat convertor in (b) a user‐friendly GUI or (c) command line. Seqformat convertor can convert between diverse types of sequence data formats

In the preset mode in EasyCodeML, likelihood‐ratio tests between nested models are performed automatically. The results are displayed on the screen at the completion of a CodeML analysis (Figure 1a). In the custom mode, likelihood‐ratio tests can also be conducted using the calculator in the utility menu of EasyCodeML (Figure 3a). We have developed a fully functional export module in the preset mode that produces a publication‐quality table containing the results of the CodeML analysis (Table 3).

**Table 3:** Example of a publication‐quality table created by the export module in EasyCodeML, based on a comparison of site models for the ECP‐EDN gene family from primates

Site model									
Model	np	Ln L	Estimates of parameters				Models compared	LRT *p*‐value	Positively selected sites
M3	15	−1,876.512700	*p*:	0.94718	0.04809	0.00473	M0 versus M3	0.00E+00	[]
			*ω*:	0.07365	8.33208	87.60345			
M0	11	−1,915.094842	*ω*_0_:	0.30409					Not allowed
M2a	14	−1,877.941799	*p*:	0.81874	0.15483	0.02642	M1a versus M2a	4.68E−07	[]
			*ω*:	0.00000	1.00000	25.64346			
M1a	12	−1,892.515966	*p*:	0.82898	0.17102				Not allowed
			*ω*:	0.00000	1.00000				
M8	14	−1,878.735192	*p*_0_ = 0.96680	*p* = 0.03134	*q* = 0.16652		M7 versus M8	9.00E−09	14 L 0.603, 133 G 0.961^*^, 228 S 0.972^*^, 230 F 0.996^**^, 231 V 0.993^**^, 232 S 0.567, 233 K 0.881, 235 D 0.953^*^, 236 G 0.996^**^, 237 G 0.973^*^, 238 R 0.999^**^, 239 Y 0.924, 279 N 0.877, 354 K 0.997^**^
			(*p*_1_ = 0.03320)	*ω* = 19.65767					
M7	12	−1,897.250798	*p* = 0.00500	*q* = 0.00637					Not allowed

Note

[], no data available.

Numerous file conversions are often required to prepare input data for CodeML. To improve the efficiency and ease of data exchange among multiple formats, we have incorporated a file‐format convertor into EasyCodeML. Named Seqformat convertor, this utility can convert CLUSTAL, FASTA, MEGA, NEXUS, and PHYLIP formats into PAML format (Figure 3b). A command‐line version of Seqformat convertor is also provided in EasyCodeML, making it possible to convert sequence formats in batch mode (Figure 3c).

We have developed a “check” module that is available for both of the running modes in EasyCodeML. The user is notified if there are discrepancies between the taxon labels in the input files (Figure S2a). This helps to satisfy the requirement of CodeML that the input sequence data and tree file have matching taxon labels.

In addition to the main functions outlined above, EasyCodeML supports parallel computation (multithreading), which is especially helpful when multiple comparisons among codon models are being performed. EasyCodeML also has drag‐and‐drop functionality for ease of use. A comparison of the features of EasyCodeML and other relevant tools or programs is provided in Table 1.

## 3 WORKED EXAMPLE

### 3.1 Preset running mode in EasyCodeML

To demonstrate the use of the clade models in the preset running mode in EasyCodeML, we present an analysis of the ECP‐EDN gene family in primates. The analyses are based on data from a study by Bielawski and Yang (2003), which investigated the role of positive selection in the evolution of this gene family.

#### 3.1.1 Step 1: Loading data and configuring parameters

EasyCodeML has two different running modes, preset and custom. In this case, we choose the preset mode (Figure 1a). We either drag‐and‐drop a folder into EasyCodeML or click on the button “…”to select a local folder as the working directory. The required inputs for analysing selection are the aligned sequences in PAML format and a tree file in Newick format. We can also drag‐and‐drop these two files into the text box. Four different model approaches are available in the preset mode. Here, we select “Clade Model” to test for positive selection in the ECP‐EDN gene family (Figure 1a).

After the sequence and tree files have been selected, press the “Check” button to check the consistency of the taxon labels between the tree and sequence files. The clade models require the nodes of the tree to be labelled in order to indicate the clades that will be assigned independent *ω* parameters, so we press the “Label” button. We then click on the entire EDN clade to be selected in the tree as the foreground lineage. The dollar symbol “$”with an integer will be shown above the EDN clade (Figure 2a). In EasyCodeML, the symbols “#”(Figure 2b) and “$”(Figure 2a) are used for the branch or branch‐site models and for the clade model, respectively.

We use other default settings for the parameters, including the “Num of Threads” and “Clean data” options. Multithreading will only take effect in the analysis using the site model. If the “Clean data” option is enabled, all sites with ambiguity characters and alignment gaps will be removed from the sequence alignment prior to analysis.

#### 3.1.2 Step 2: CodeML analysis

Before starting the CodeML analysis, we need to click on the “Save Current Profile” button to enable all parameters for the current analysis. The button “Run CodeML” then starts the CodeML analysis. At the conclusion of the analysis, the log‐likelihood (lnL) values and the number of parameters (np) will be automatically retrieved. A likelihood‐ratio test is performed for the nested models and all results are automatically organized and displayed on the screen (Figure 1a).

#### 3.1.3 Step 3: Summarizing and interpreting results

A publication‐quality table that contains all of the relevant information from the CodeML analyses can be generated using the “Export” button. Microsoft Excel can be launched to view the saved results file by clicking on “View”. A clear rejection of the null model indicates that divergent selection was detected between the foreground (the entire EDN clade) and background branches (the entire ECP clade). Note that the selection analysis presented here is merely instructional. If there are suboptimal peaks in the likelihood surface, we can load and edit the control file in the custom running mode in EasyCodeML, and then run the program several times to find the globally optimal likelihood score using different initial values of *ω*.

### 3.2 Custom running mode in EasyCodeML

We briefly illustrate the use of the custom running mode in EasyCodeML by analysing a data set from Padhi, Verghese, and Otta (2009). We compare the M8 and M8a models to test for sites under positive selection in the outer membrane protein C (*ompC*) of strains of *Enterobacter aerogenes*, although this particular model comparison is also available in the preset running mode of EasyCodeML.

#### 3.2.1 Step 1: Loading data and configuring parameters

We switch current running mode to the custom mode and specify a local folder as the working directory using drag‐and‐drop, as described above for the preset mode. The “Load” button can be used to load a codon model available from a control file viewer (Supporting information Figure S2b). This will bring up a dialogue box from which we choose the M8a model. We can further modify the various parameter values to meet different requirements. Tree labelling is necessary when examining the branch‐related models (branch models, branch‐site models, and clade models), but not with the site models. Therefore, default values are used for all parameters except for leaving “Clean data” unchecked (Figure 1b). We need to save the current profile using “Save Current Profile” after checking whether the taxon labels match between the tree and sequence files.

#### 3.2.2 Step 2: CodeML analysis

Clicking “Run CodeML” will start the analysis. In order to perform the subsequent likelihood‐ratio test, we will need to run both models. Therefore, we need to repeat the procedure for the M8 model.

We navigate to the working directory and locate the main result files (mlc) of the model M8 and M8a. After noting the log‐likelihood (lnL) values and the number of parameters (np) in these mlc files, we enter them in the LRT calculator from the “Tools” menu and run a likelihood‐ratio test. Based on the lnL and np values of the null model (M8, lnL = −1878.7, np = 14) and the alternative model (M8a, lnL = −1,892.5, np = 13), the test yields a p‐value below 0.05 (Figure 3a).

#### 3.2.3 Step 3: Identifying sites under selection

In the comparison of models M8 and M8a, the BEB analysis under model M8 is used to identify codons under positive selection. Thus, we find a block called “Bayes Empirical Bayes (BEB) analysis” in the mlc file (Supporting information Figure S3). This block lists the amino acids that have a BEB score higher than 0.5. Sites potentially under positive selection are suggested by BEB values higher than 0.95, which are indicated by asterisks. In this data set, we identified nine codons as being under positive selection with posterior probability >0.95, matching the results of Padhi et al. (2009).

## 4 CONCLUSIONS

We have developed EasyCodeML, an interactive visual tool for analyses of selection that incorporates the major codon‐based models in CodeML. EasyCodeML includes a feature that allows interactive labelling of the tree in branch‐ or clade‐specific analyses. We hope that the program proves to be a useful tool for studies of molecular evolution, by broadening the user base of CodeML and improving its usability. EasyCodeML is an ongoing project and we welcome bug reports, feedback, and suggestions.

## CONFLICT OF INTEREST

None declared.

## AUTHOR CONTRIBUTIONS

F. Gao and C. Chen conceived the idea, developed the program, and led the writing of the manuscript. D. A. Arab, Z. Du, Y. He, and S.Y.W. Ho contributed to the manuscript.

## DATA ACCESSIBILITY

The stand‐alone package and user manual of EasyCodeML are hosted on GitHub: https://github.com/BioEasy/EasyCodeML.
