# Supplementary figures and images for: EasyCodeML: A visual tool for analysis of selection using CodeML
Source: Ecol Evol. 2019 Mar 1;9(7):3891–8. doi: 10.1002/ece3.5015 (PMC6467853; doi:10.1002/ece3.5015)

Figure S1

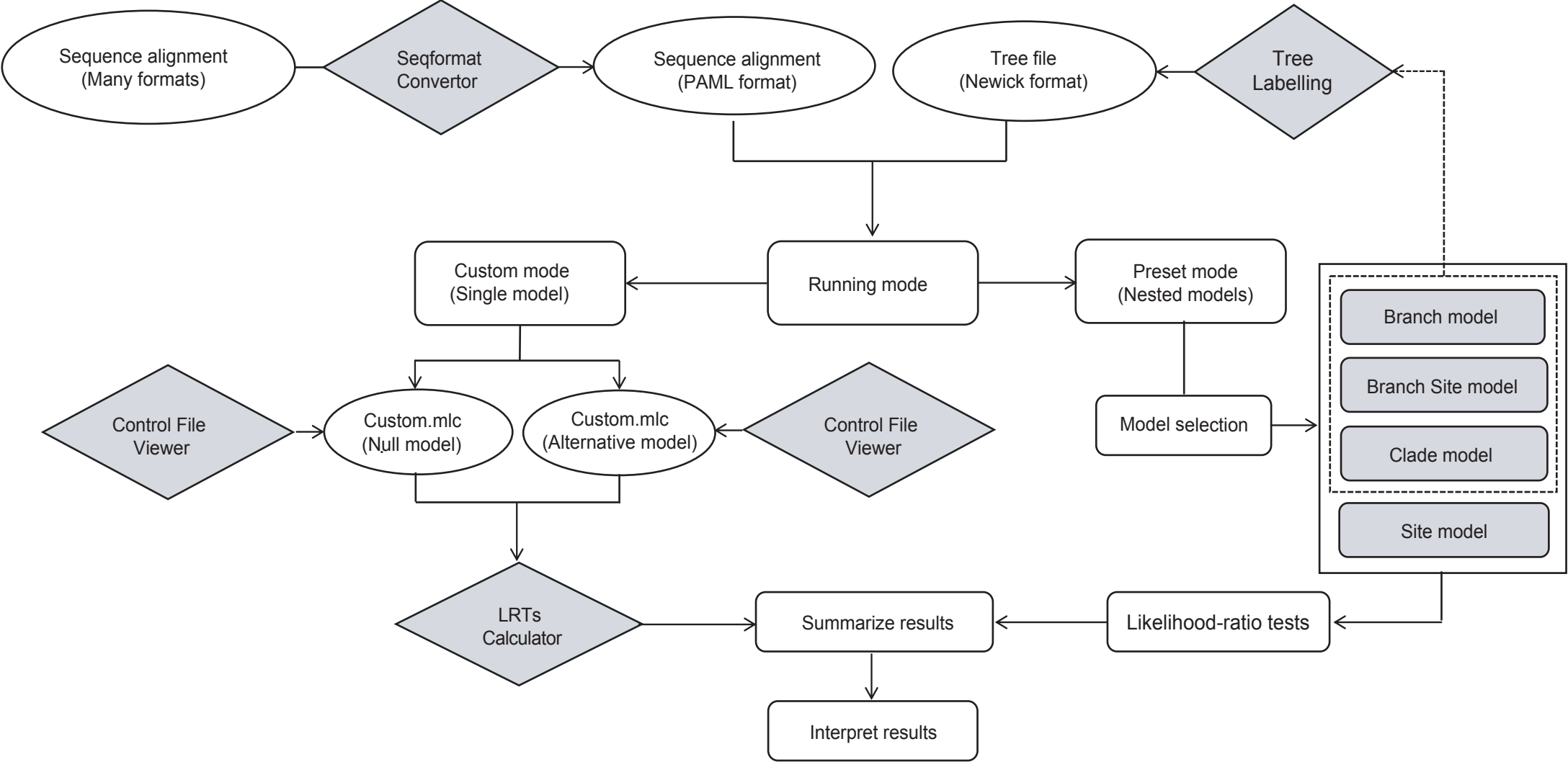

Supplement: Supplementary file 1 [file ECE3-9-3891-s001.pdf]

Figure S2

(a).

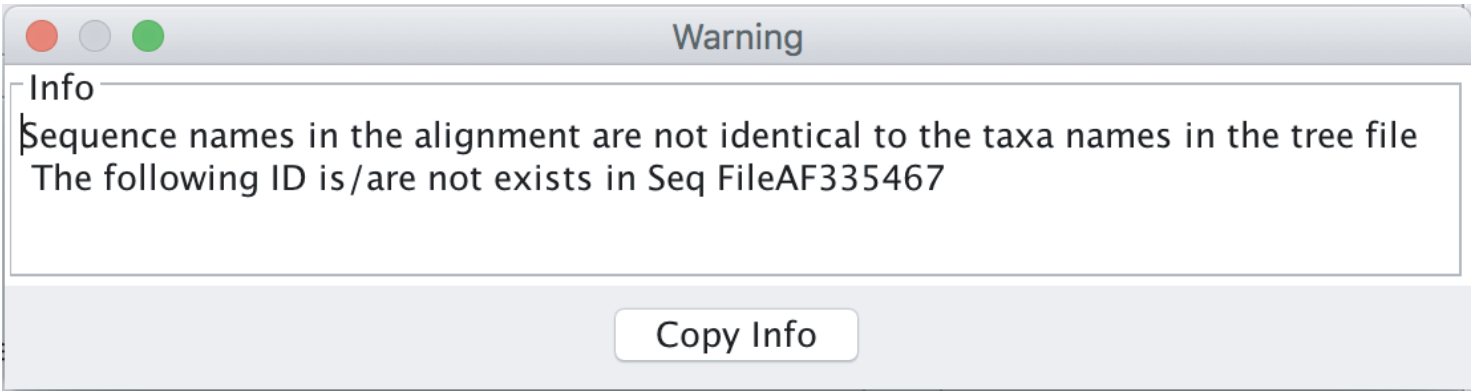

(b).

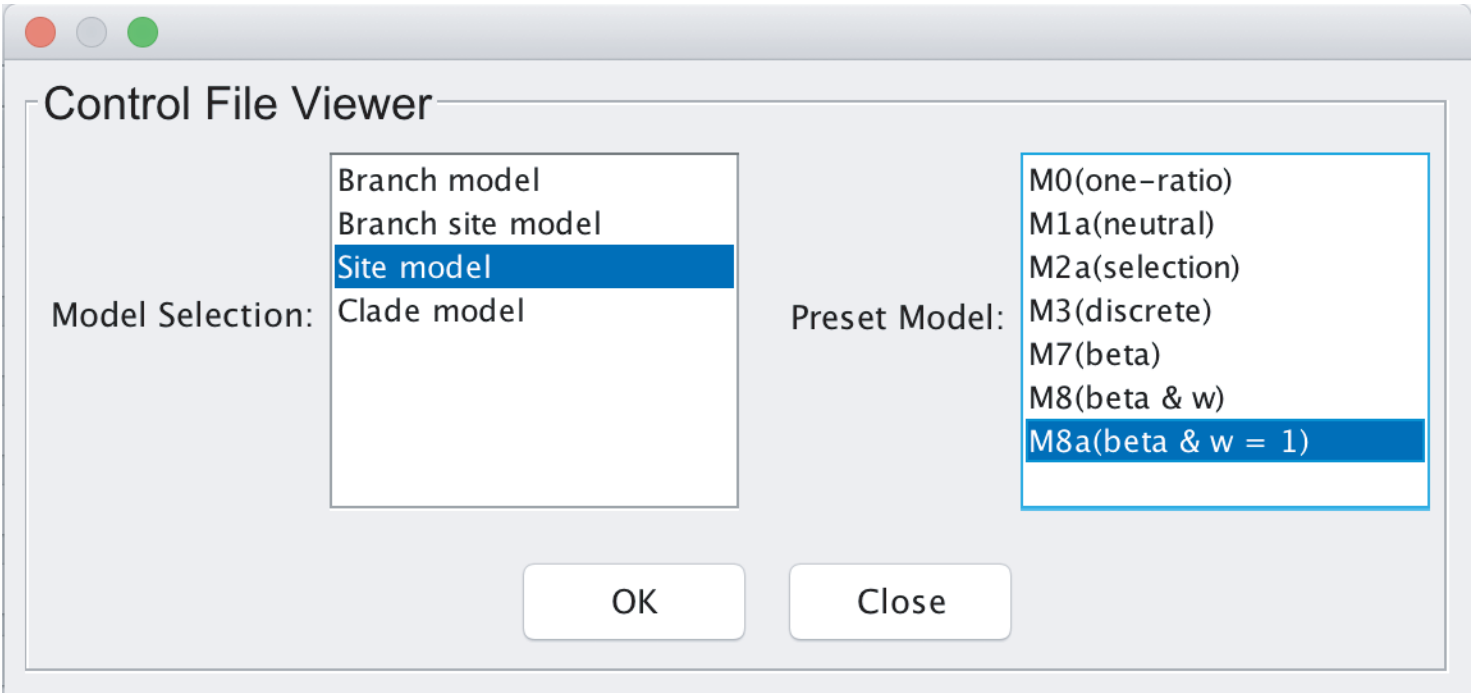

Supplement: Supplementary file 2 [file ECE3-9-3891-s002.pdf]
