# Supplementary material for: EasyCodeML: A visual tool for analysis of selection using CodeML
Source: Ecol Evol. 2019 Mar 1;9(7):3891–8. doi: 10.1002/ece3.5015 (PMC6467853; doi:10.1002/ece3.5015)
Supplement: Supplementary file 3 [file ECE3-9-3891-s003.pdf]

Figure S3

```
Bayes Empirical Bayes (BEB) analysis (Yang, Wong & Nielsen 2005. Mol. Biol. Evol. 22:1107-1118)
Positively selected sites (*: P>95%; **: P>99%)
(amino acids refer to 1st sequence: AF335467)

            Pr(w>1)     post mean +- SE for w

    14 L      0.603         5.746 +- 4.508
   133 G      0.961*        8.984 +- 2.082
   229 S      0.972*        9.080 +- 1.891
   231 F      0.996**       9.295 +- 1.329
   232 V      0.993**       9.264 +- 1.427
   233 S      0.566         5.411 +- 4.553
   234 K      0.881         8.272 +- 3.058
   236 D      0.953*        8.915 +- 2.210
   237 G      0.996**       9.293 +- 1.334
   238 G      0.973*        9.091 +- 1.866
   239 R      0.999**       9.317 +- 1.256
   240 Y      0.925         8.663 +- 2.596
   280 N      0.877         8.240 +- 3.094
   355 K      0.997**       9.295 +- 1.326
```
