# Supplementary material for: EasyCodeML: A visual tool for analysis of selection using CodeML
Source: Ecol Evol. 2019 Mar 1;9(7):3891–8. doi: 10.1002/ece3.5015 (PMC6467853; doi:10.1002/ece3.5015)
Supplement: Supplementary file 4 [file ECE3-9-3891-s004.pdf]

TABLE S1 The control file parameters values defined for the branch models in EasyCodeML

Parameter	Branch model		
	**M0**	**Two-ratio**	**Free ratio**
seqfile	User	User	User
treefile	User	User	User
outfile	M0_mlc	BM_mlc	FM_mlc
noisy	9	9	9
verbose	0	0	1
runmode	0	0	0
seqtype	1	1	1
CodonFreq	2	2	2
clock	0	0	0
aaDist	0	0	0
model	0	2	1
Nssites	0	0	0
icode	0	0	0
Mgene	0	0	0
fix_kappa	0	0	0
kappa	2	2	2
fix_omega	0	0	0
omega	2	2	1
fix_alpha	1	1	1
alpha	0	0	0
Malpha	0	0	0
ncatG	3	3	4
getSE	0	0	0
RateAncestor	0	0	0
fix_blength	0	0	0
method	0	0	0
Small_Diff	.45e-6	.45e-6	.45e-6
cleandata	1	1	1

TABLE S2 The control file parameters values defined for the branch-site models in EasyCodeML

Parameter	Branch-site model	
	**Model A**	**Model A_{null}**
seqfile	User	User
treefile	User	User
outfile	A_mlc	Anull_mlc
noisy	9	9
verbose	0	0
runmode	0	0
seqtype	1	1
CodonFreq	2	2
clock	0	0
aaDist	0	0
model	2	2
Nssites	2	2
icode	0	0
Mgene	0	0
fix_kappa	0	0
kappa	2	2
fix_omega	0	1
omega	2	1
fix_alpha	1	1
alpha	0	0
Malpha	0	0
ncatG	3	3
getSE	3	0
RateAncestor	0	0
fix_blength	0	0
method	0	0
Small_Diff	.45e-6	.45e-6
cleandata	1	1

TABLE S3 The control file parameters values defined for the site models in EasyCodeML

Parameter	Site model						
	**M0**	**M1a**	**M2a**	**M3**	**M7**	**M8**	**M8a**
seqfile	User	User	User	User	User	User	User
treefile	User	User	User	User	User	User	User
outfile	M0_mlc	M1a_mlc	M2a_mlc	M3_mlc	M7_mlc	M8_mlc	M8a_mlc
noisy	9	9	9	9	9	9	9
verbose	0	0	0	0	0	0	0
runmode	0	0	0	0	0	0	0
seqtype	1	1	1	1	1	1	1
CodonFreq	2	2	2	2	2	2	2
clock	0	0	0	0	0	0	0
aaDist	0	0	0	0	0	0	0
model	0	0	0	0	0	0	0
Nssites	0	1	2	3	7	8	8
icode	0	0	0	0	0	0	0
Mgene	0	0	0	0	0	0	0
fix_kappa	0	0	0	0	0	0	0
kappa	2	2	2	2	2	2	2
fix_omega	0	0	0	0	0	0	1
omega	2	2	2	2	2	2	1
fix_alpha	1	1	1	1	1	1	1
alpha	0	0	0	0	0	0	0
Malpha	0	0	0	0	0	0	0
ncatG	3	3	3	3	3	3	3
getSE	0	0	0	0	0	0	0
RateAncestor	0	0	0	0	0	0	0
fix_blength	0	0	0	0	0	0	0
method	0	0	0	0	0	0	0
Small_Diff	.45e-6	.45e-6	.45e-6	.45e-6	.45e-6	.45e-6	.45e-6
cleandata	1	1	1	1	1	1	1

TABLE S4 The control file parameters values defined for the clade models in EasyCodeML

Parameter	Clade model	
	**CmC**	**M2a_rel**
seqfile	User	User
treefile	User	User
outfile	CmC_mlc	M22_mlc
noisy	9	9
verbose	1	1
runmode	0	0
seqtype	1	1
CodonFreq	2	2
clock	0	0
aaDist	0	0
model	3	0
Nssites	2	22
icode	0	0
Mgene	0	0
fix_kappa	0	0
kappa	3	3
fix_omega	0	0
omega	1	1
fix_alpha	1	1
alpha	0	0
Malpha	0	0
ncatG	10	10
getSE	1	1
RateAncestor	1	1
fix_blength	-1	-1
method	0	0
Small_Diff	.5e-6	.5e-6
cleandata	1	1
